# Supplementary material for: Main and interacting effects of physical activity and sedentary time on older adults’ BMI: The moderating roles of socio-demographic and environmental attributes
Source: PLoS One. 2020 Jul 9;15(7):e0235833. doi: 10.1371/journal.pone.0235833 (PMC7347204; doi:10.1371/journal.pone.0235833)
Supplement: Attachment — Submitted filename: Comments to editor.doc [file pone.0235833.s002.doc]

In the current manuscript, Dyck et al assessed the main and interacting effects of PA and ST with BMI and overweight/obese in Hong Kong and Ghent older adults. They found that ST was linearly and positively related to weight outcomes in Hong Kong and Belgian older adults, while MVPA was not. Although this study have important public health implications, but there are some limitations to the study. The manuscript could be strengthened by addressing the following issues and then reconsidering the potential for publication:

1. The author found the positive association between ST and BMI was significantly stronger at lower than higher values of MVPA in Hong Kong older adults but an opposite trend in Belgian population. Table 1 should compares baseline characteristics of the study participants from Hong Kong and those from Ghent using relevant statistical methods. Knowing differences between these groups helps the reader understand the results of this article.

2. What is the explanation for why the study participants in Hong Kong were mostly women?

3. The author adopted a targeted obesity standard for the Hong Kong population, is there a targeted obesity standard for the Belgian population?

4. A large proportion of the older adults, both in Hong Kong (90.0%) and in Ghent (68.9%) reached the health guideline of 150 minutes of MVPA per week. The author could divide these participants into moderate physical activity group and heavy physical activity group to further explore the association of ST and PA with weight outcomes.

5. Please rephrase with a focus on the public health implications of your findings in terms of its practical utility taking current practices and clinical feasibility into account in Conclusions.
